# Unequal socioeconomic distribution of the primary care workforce: whole-population small area longitudinal study

**DOI:** 10.1136/bmjopen-2015-008783

**Published:** 2016-01-12

**Authors:** Miqdad Asaria, Richard Cookson, Robert Fleetcroft, Shehzad Ali

**Affiliations:** 1 Centre for Health Economics, University of York, York, UK; 2 Norwich Medical School, University of East Anglia, Norwich, UK; 3 Department of Health Sciences, University of York, York, UK

**Keywords:** PRIMARY CARE, Health Workforce, Health Inequality

## Abstract

**Objective:**

To measure changes in socioeconomic inequality in the distribution of family physicians (general practitioners (GPs)) relative to need in England from 2004/2005 to 2013/2014.

**Design:**

Whole-population small area longitudinal data linkage study.

**Setting:**

England from 2004/2005 to 2013/2014.

**Participants:**

32 482 lower layer super output areas (neighbourhoods of 1500 people on average).

**Main outcome measures:**

Slope index of inequality (SII) between the most and least deprived small areas in annual full-time equivalent GPs (FTE GPs) per 100 000 need adjusted population.

**Results:**

In 2004/2005, inequality in primary care supply as measured by the SII in FTE GPs was 4.2 (95% CI 3.1 to 5.3) GPs per 100 000. By 2013/2014, this SII had fallen to −0.7 (95% CI −2.5 to 1.1) GPs per 100 000. The number of FTE GPs per 100 000 serving the most deprived fifth of small areas increased over this period from 54.0 to 60.5, while increasing from 57.2 to 59.9 in the least deprived fifth, so that by the end of the study period there were more GPs per 100 000 need adjusted population in the most deprived areas than in the least deprived. The increase in GP supply in the most deprived fifth of neighbourhoods was larger in areas that received targeted investment for establishing new practices under the ‘Equitable Access to Primary Medical Care’.

**Conclusions:**

There was a substantial reduction in socioeconomic inequality in family physician supply associated with national policy. This policy may not have completely eliminated socioeconomic inequality in family physician supply since existing need adjustment formulae do not fully capture the additional burden of multimorbidity in deprived neighbourhoods. The small area approach introduced in this study can be used routinely to monitor socioeconomic inequality of access to primary care and to indicate workforce shortages in particular neighbourhoods. http://creativecommons.org/licenses/by/4.0

**Strengths and limitations of this study**

- Our study introduces a new small area level method for measuring inequality in general practitioner supply that focuses specifically on socioeconomic inequality and captures inequality within National Health Service (NHS) administrative areas as well as between them.
- The main limitation of this study is the lack of a generally accepted and up-to-date measure of relative need for primary care in deprived small areas.
- Currently, the best available measure is the workload adjustment recommended in the 2007 review of the Carr-Hill formula for allocating primary care funding. However, concerns have been raised that the Carr-Hill formula may not fully reflect the additional needs for primary care in deprived populations.

## Introduction

There is long-standing international policy concern about unequal socioeconomic distribution of the primary care workforce, which can harm population health and contribute to wider socioeconomic inequalities in health.^1–3^ As the UK Chair of the Royal College of General Practitioners recently wrote, “The general practice workforce is unevenly spread across the country, with the fewest doctors in the most deprived areas, exacerbating health inequalities”.^4^ This problem may grow in future, as substantial future primary care workforce shortages are projected over the next two decades in the UK, USA and elsewhere.^4–6^ Demand for primary care is increasing due to increasing numbers of people with multiple chronic conditions (multimorbidity), especially in deprived populations,^7–9^ and attempts by policymakers to shift care from secondary to primary care settings.^10^ Workload is also increasing due to the increasing complexity of care and associated administrative burdens.^11^ In England, for example, the Royal College of General Practitioners estimates that 8000 more full-time equivalent (FTE) primary care physicians (general practitioners (GPs)) will be needed by 2020,^12^ while worryingly recent trends indicate a fall in applications for medical training in primary care.^13^

Previous studies have found substantial geographical inequalities in family physician supply between large subnational areas, even in high-income countries with universal health coverage.^14–21^ However, because these studies have focused on large areas they have not been able to accurately describe socioeconomic inequality in primary care supply by pinpointing primary care shortages in specific disadvantaged neighbourhoods. Studies in England using data from 1974 to 2006 have found substantial and persistent geographical inequality in GP supply relative to need between National Health Service (NHS) administrative areas—Family Practitioner Committees until 1990, then Family Health Service Authorities until 2000, then Primary Care Trusts (PCTs).^22–26^ Historically, these inequalities have been largely impervious to NHS policy initiatives designed to reduce them, such as the deprivation-weighted capitation payments introduced in 1990. There is also evidence that some policies may have increased large area inequality, such as the abolition of entry controls in ‘overdoctored’ areas in England in 2002.^22^

In the late 2000s following the 2006 White Paper ‘Our Health, Our Care, Our Say’, a renewed effort was made to increase GP supply in deprived areas as part of wider attempts to meet government targets for reducing health inequality.^24^ ^27–29^ Most notably, the ‘Equitable Access to Primary Medical Care’ (EAPMC) programme that invested £250 million towards establishing new general practices and GP-led heath centres as well as extending opening hours and expanding services in the 38 most ‘underdoctored’ PCT areas.^28^ This programme was announced by a Labour government in the 2006 White Paper, funded from 2008,^28^ and wound down from 2011, a year or so after the new Coalition government came to power.^30^ Our study aims to measure socioeconomic inequality in GP supply from 2004/2005 to 2013/2014, and to examine whether the EAPMC programme was associated with any beneficial impact on reducing socioeconomic inequality. Our study introduces a new way of measuring inequality in GP supply, based on small area variations, which focuses specifically on socioeconomic inequality. Studies based on large area variations may mask important changing patterns of socioeconomic inequality within administrative areas. Our study examines variation between small area populations of approximately 1500 people, allowing us to capture changing patterns of socioeconomic inequality in much more fine-grained detail than previous studies.

## Data and methods

We constructed whole-population national data sets at both small area level and practice level. Using the NHS Attribution Data Set of GP-registered populations, we linked practice level data on primary care supply for the 10 years, 2004/2005 through 2013/2014, with corresponding small area level data on population and deprivation. We use data from all 9092 general practices in the English NHS that were open for at least 1 year of the study period. Our data on primary care supply were obtained from the annual NHS General and Personal Medical Services workforce census, taken at 30 September each year, midway through the financial year.

In line with previous research studies and official reports, the primary indicator of GP supply reported in this study is the FTE number of GP principals and salaried GPs, who make up the vast majority of the GP workforce.^4^ ^22^ ^23^ ^27^ ^31^ We also conducted robustness checks using other GP supply variables, including (1) headcount of GP principals and salaried GPs; (2) GP registrars (trainee doctors on short-term placements having ‘supernumerary’ contracts, designed primarily for training rather than delivering patient care);^32^ and (3) GP retainers (sessional GPs who only work a maximum of four sessions of approximately half a day each week, and only make up a small fraction of the workforce).^33^ ^34^ We also conducted robustness checks using the limited available data on practice nurse supply, available at practice level for 2013/2014 but only at PCT level before that. Our data do not include locum GPs or supply of emergency primary care services outside normal office hours.

The small area unit of analysis was the 2001 lower super output area (LSOA)—a geographical unit defined by the 2001 census. There are 32 482 of these small areas in England each with a mean population of approximately 1500 people. Data on the LSOA of residence of each practice-registered patient for each year were used to attribute GP supply from practice level to LSOA level, using population-weighted averages. LSOAs were ranked by deprivation according to their Index of Multiple Deprivation (IMD) 2010 ranks, and split into deprivation quintile and decile groups with equal numbers of LSOAs in each group. Office for National Statistics (ONS) mid-year population estimates at LSOA level were used to derive the population of each deprivation group. We used ONS population estimates because GP practice list data are less thoroughly cleaned and validated and tends to overestimate population size, for example, due to people leaving the area without notifying their GP. LSOA populations were adjusted for their relative needs for primary care using the workload adjustment aspect of the most recently updated version of the Carr-Hill formula for primary care resource allocation.^35^ This version of the formula was recommended in 2007 by the Formula Review Group established by NHS employers and the British Medical Association (BMA), and though never implemented in practice it remains the most authoritative and up-to-date analysis of the determinants of primary care workload in England. This adjustment takes into consideration the age and sex structure and IMD health deprivation score of each LSOA to upscale populations that are expected to require more primary care and downscale populations expected to require less. We report both adjusted and unadjusted results, and also conduct robustness checks using an alternative need formula: the 2013/2014 Nuffield index of general and acute hospital need.^36^ As a further robustness check, the analysis was repeated at practice level by reverse attributing LSOA population and deprivation variables to GP practices and aggregating GP supply numbers by population-weighted practices into five approximately equally sized deprivation-based groups. To provide insight into the components of change in GP supply, we also produced descriptive statistics by deprivation group and year on the numbers of practices opening and closing, the average size of GP practices, and the average number of small areas served by each practice as an indication of whether increases in GP supply can be attributed to patients travelling further.

The primary measures of inequality were the slope index of inequality (SII) and relative index of inequality (RII), both based on linear regression analysis at the level of IMD decile group. This involves modelling GP supply as a linear function of deprivation decile, entered as a continuous variable scaled from 0 to 1. The SII is the coefficient in this regression; the RII is that coefficient divided by the mean GP supply. The SII can be interpreted as the modelled difference in the number of FTE GPs per 100 000 population between the most and least deprived small areas (the absolute gap); while the RII can be interpreted as this difference as a proportion of the national average (the proportionate gap). Regression models using pooled data for multiple years were used to test whether observed changes in inequality between years were statistically significant, based on interaction terms between year and deprivation.

To examine associations between change in GP supply inequality and the EAPMC programme, we identified the 38 PCTs that were considered to be ‘underdoctored’ and hence eligible to receive funding from this programme from a Department of Health press release on the policy.^37^ We then compared changes in GP supply by deprivation group of LSOAs within these ‘underdoctored’ PCTs (which cover a population of approximately 10 million people) with changes in GP supply in deprivation groups of LSOAs within the remaining PCTs (which cover a population of approximately 43 million people), focusing on change between the year the policy was announced, in 2006, and the year the policy was wound down, in 2011.

## Results

**Figure 1:**
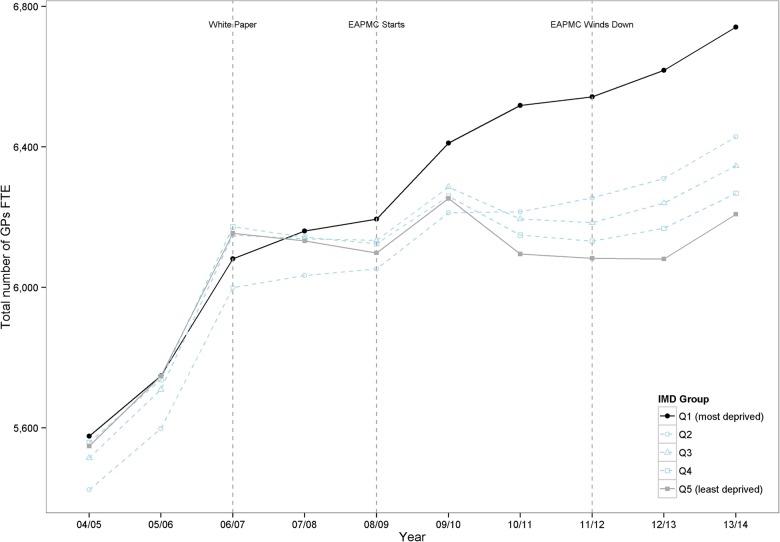
Total GP workforce1 by Deprivation Quintile Group, from 2004/2005 to 2013/2014. Note: Number of FTE GPs, excluding registrars and retainers. FTE, full time equivalent; GP, general practitioner; IMD, Index of Multiple Deprivation.

Total numbers of GPs in England by year are reported in table 1, in terms of both headcount and FTE, along with total population figures. Although the total headcount of GPs continued to increase throughout the period, FTE numbers have been approximately flat since 2009/2010 while the patient population has continued to grow. In England as a whole, GP supply increased from 55.1 to 60.2 FTE GPs per 100 000 population from 2004/2005 to 2006/2007, but remained approximately stable thereafter, rising to 60.7 in 2009/2010 then falling to 59.4 by 2013/2014. Crude trends in total numbers of FTE GPs split by small area level deprivation are shown in figure 1 (these are not adjusted for population change). Total numbers of FTE GPs have grown much faster in the most deprived fifth of English small areas than elsewhere, with GP supply in the most affluent fifth growing at the slowest pace over the past 10 years. This pattern is also reflected in the raw headcount of GPs (see online supplementary appendix figure A4.3).

**Table 1:** Total GP workforce in England from 2004/2015 to 2013/2014*

		GP headcount		GP full-time equivalent	
Year	Total population	Total	Per 100 000 population	Total	Per 100 000 population
2004/2005	50 109 707	30 751	61.37	27 621	55.12
2005/2006	50 466 162	31 924	63.26	28 540	56.55
2006/2007	50 763 893	32 646	64.31	30 557	60.19
2007/2008	51 106 181	32 995	64.56	30 609	59.89
2008/2009	51 464 646	33 911	65.89	30 603	59.46
2009/2010	51 807 127	35 072	67.70	31 422	60.65
2010/2011	52 234 045	36 073	69.06	31 173	59.68
2011/2012	52 690 703	36 628	69.52	31 197	59.21
2012/2013	53 488 001	36 771	68.75	31 418	58.74
2013/2014	53 859 917	36 849	68.42	31 993	59.40

*Excluding GP registrars, retainers and locums.

GP, general practitioner.

**Figure 2:**
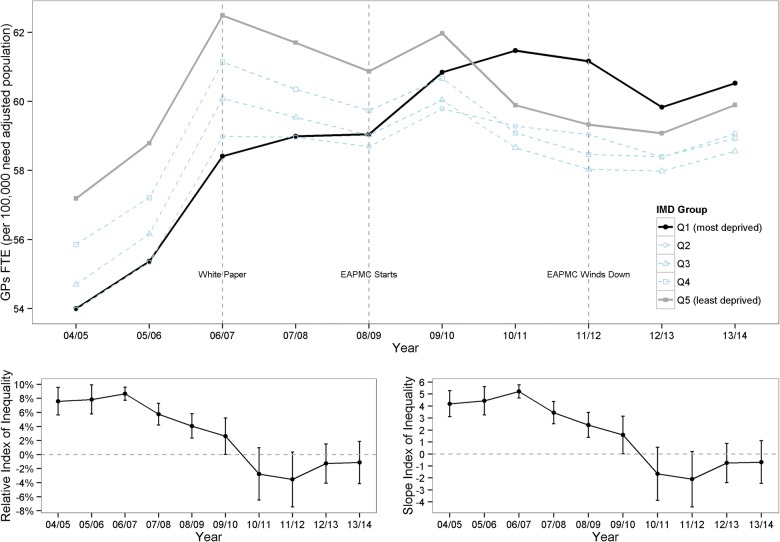
Socioeconomic inequality in GP supply in England 2003/2004 to 2013/2014. Note: (1). The upper panel shows FTE GPs per 100 000 need adjusted population by deprivation quintile group of small areas by year; the two lower panels show inequality indices by year, with 95% CIs. (2). The slope index of inequality can be interpreted as the absolute gap in FTE GPs per 100 000 need adjusted population between the most and least deprived small area, and the relative index of inequality as the percentage gap relative to the average area. In each case, a positive index indicates ‘prorich’ inequality favouring less deprived areas. EAPMC, Equitable Access to Primary Medical Care; FTE, full time equivalent; GP, general practitioner; IMD, Index of Multiple Deprivation.

Figure 2 shows these trends adjusted for population size and need. In England as a whole, GP supply increased relative to population need from 2004/2005 to 2006/2007 but remained approximately stable thereafter. The geographical distribution of this GP supply in relation to the deprivation of the areas served by GPs, however, changed substantially over the study period. In 2004/2005, there was ‘prorich’ inequality in GP supply relative to need, with 54.0 FTE GPs per 100 000 of need adjusted population in the most deprived fifth of small areas and 57.2 FTE GPs per 100 000 of need adjusted population in the least deprived fifth of areas resulting in an SII of 4.2 (95% CI 3.1 to 5.3). By the end of the study period, this inequality had reversed with 60.5 and 59.9 FTE GPs per 100 000 need adjusted population in the most deprived and least deprived fifths of small areas, respectively, and an SII of −0.7 (95% CI −2.5 to 1.1).

This decrease in socioeconomic inequality in GP supply relative to need occurred between 2006/2007 and 2011/2012, a period over which the SII fell from 5.2 (95% CI 4.7 to 5.8) to −2.1 (95% CI −4.4 to 0.2). During this 5-year period, people living in the most deprived fifth of English small areas experienced a steady increase in GP supply relative to need, which was particularly rapid from 2008/2009 to 2010/2011, while people living in the least deprived three-fifths experienced a decline. By 2010/2011, the ‘prorich’ inequality in GP supply relative to need appeared to have disappeared. Nationally, the increase in GP supply relative to need in deprived small areas from 2006/2007 to 2011/2012 was offset by a corresponding reduction in other areas—resulting in a slight overall decline in national GP supply relative to need from 60.2 to 59.2 FTE GPs per 100 000. These inequality trends were driven largely by change in the most and least deprived quintile groups: GP supply in the middle three quintile groups changed little, and remained lower than in the most affluent quintile group.

By 2013/2014, the trend in GP supply per need weighted population appeared to have reversed with GP supply in the most affluent areas growing faster than in the most deprived areas.

**Figure 3:**
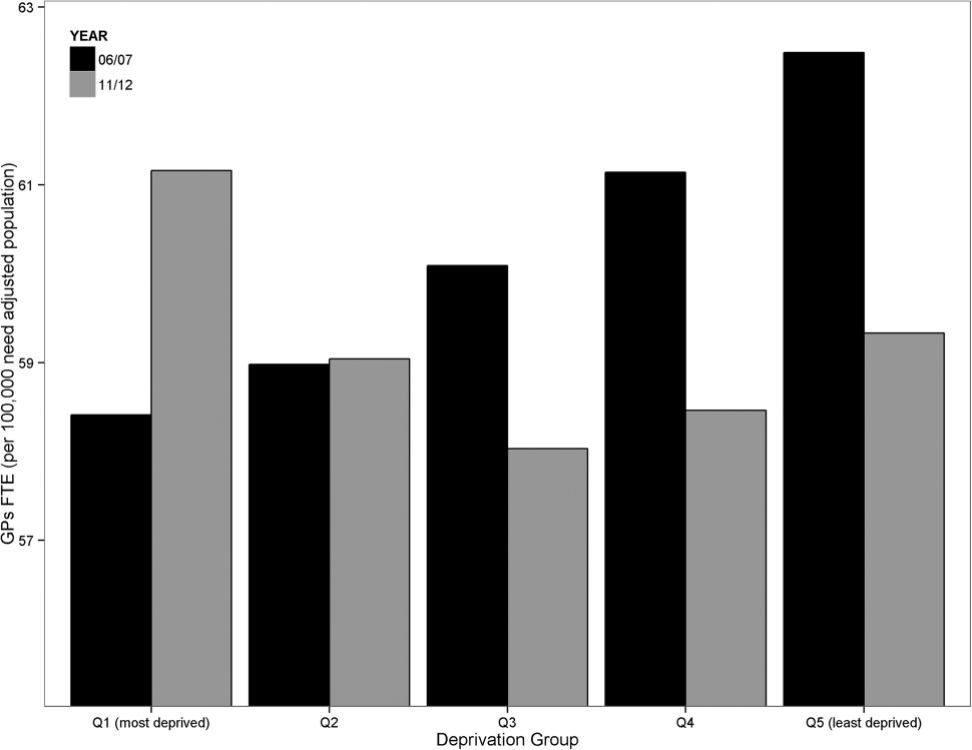
Socioeconomic gradient in GP supply in 2006/2007 and 2011/2012, before and after the Equitable Access to Primary Medical Care programme. FTE, full time equivalent; GP, general practitioner.

Cross-sectional results for 2006/2007 and 2011/2012, before and after the EAPMC programme, are presented in figure 3. This highlights the reversal of the gradient in GP supply from favouring the least deprived areas in 2006/2007 to favouring the most deprived areas in 2011/2012.

**Figure 4:**
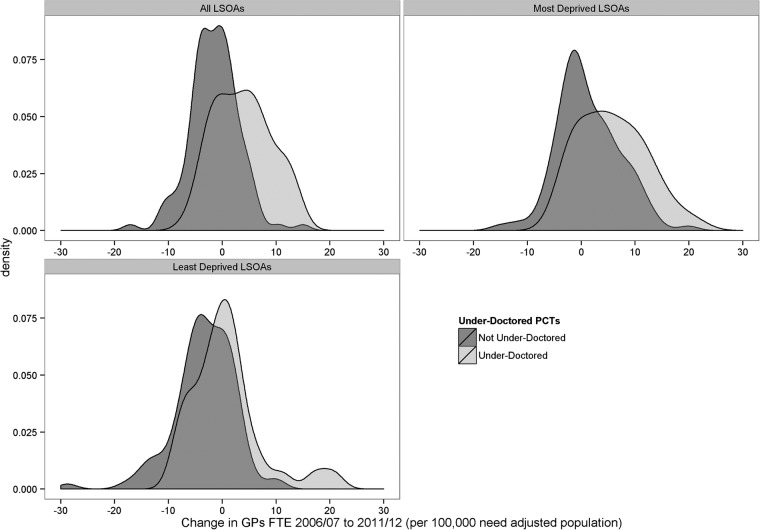
Change in GP supply between 2006/2007 and 2011/2012 by Deprivation Quintile Group, comparing ‘underdoctored’ PCTs and other PCTs (Kernel density plots). FTE, full time equivalent; GP, general practitioner; LSOA, lower super output area; PCT, Primary Care Trust.

Figure 4 shows changes in GP supply between these years, comparing LSOAs in ‘underdoctored’ PCTs that received funding under the EAMPC programme with those in the other PCTs that did not receive this funding. PCTs classified as ‘underdoctored’ experienced larger increases in GP supply than PCTs not classified as ‘underdoctored’. Furthermore, these larger increases were concentrated in the poorest fifth of LSOAs in England.

The reduction in the SII between 2006/2007 and 2011/2012 when measured at LSOA level (average population 1500) was 7.3 (95% CI 4.9 to 9.7). The same reduction in SII when measured at the much larger CCG level (average population 250 000) was 6.9 (95% CI 1.7 to 12.1). The greater value of the change in SII found when using the finer grained geography demonstrates that by conducting our analysis at the small area level, we are able to identify both changes in within CCG inequality as well as changes in between CCG inequality, the first of which would have been overlooked had the analysis been conducted at the larger unit of analysis.

Our main finding of a reduction in socioeconomic inequality in GP supply from 2006/2007 to 2011/2012 was robust to extensive sensitivity analyses using different definitions of primary care supply (headcount and FTE, with and without adjustment for population size (see online supplementary appendix figure A4.3) and need (see online supplementary appendix figure A4.4), with and without GP registrars and retainers (see online supplementary appendix figures A4.1 and A5.1), with and without practice nurses at PCT level (see online supplementary appendix figures A14.1 and A14.3), different units of analysis small area (see online supplementary appendix figure A4.1), practice (see online supplementary appendix figure A8.1), PCT (see online supplementary appendix figure A14.1) and CCG (see online supplementary appendix figure A15.1) and different measures of inequality (absolute and relative)). This finding was also robust to using a different need adjustment formula: the Nuffield general and acute hospital need index for 2013/2014 (see online supplementary appendix figure A17.3).^36^

The greater increase in GP supply in deprived small areas appears primarily to have been driven by the opening of new practices, rather than recruitment into existing practices. In 2009/2010, 2010/2011 and 2011/2012, there were substantial net increases in GP supply in deprived areas of around 28, 167 and 26 FTE GPs, respectively, resulting from the opening and closing of practices (see online supplementary appendix table 1.7). However, this was followed by substantial net falls in both subsequent years of around 55 and 65 FTE GPs, respectively, as more practices closed than opened. Meanwhile, average practice size grew at similar rates in all deprivation groups (see online supplementary appendix figure 8.6). There does not appear to be any evidence of patients living in deprived areas travelling further to increase their access to GPs, on the contrary average numbers of LSOAs per practice remained stable throughout the 10-year period of the study (see online supplementary appendix figure 8.5). Full details of these results as well as further breakdowns of the results presented in the paper can be found in the accompanying online supplementary appendix.

## Discussion

### Statement of principal findings

We found a substantial reduction in socioeconomic inequality in GP supply in England from 2006/2007 to 2011/2012. This can partly be attributed to national policy in the form of the EAPMC programme, which provided additional funding for new GP practices in ‘underdoctored’ areas of the country. The greater increase in GP supply in deprived small areas appears primarily to have been driven by the opening of new practices, rather than recruitment into existing practices. Socioeconomic inequality in GP supply subsequently increased slightly in 2012/2013 and 2013/2014, as the NHS funding situation tightened and practices started closing more rapidly in deprived areas.

### Strengths and weaknesses of the study

Our study introduces a new small area level method for measuring inequality in GP supply that focuses specifically on socioeconomic inequality and captures inequality within NHS administrative areas as well as between them. Previous large area level methods can only tell policymakers which Clinical Commissioning Groups (CCGs) are the most ‘underdoctored’. As well as this, our new method also allows policymakers to take a close-up look at the situation within CCGs and identify which individual neighbourhoods and GP practices are the most deprived and underdoctored. This ability could potentially be used to redirect funding for new practices and new GPs more accurately towards the neighbourhoods that need them most.

The main limitation of this study is the lack of a generally accepted and up-to-date measure of relative need for primary care in deprived small areas. Currently, the best available measure is the workload adjustment recommended in the 2007 review of the Carr-Hill formula for allocating primary care funding.^35^ This adjustment is based on regression analysis of the determinants of consultation rates in a sample of 454 practices serving 3.8 million patients from April 2003 to April 2004.^38^ However, concerns have been raised that the Carr-Hill formula may not fully reflect the additional needs for primary care in deprived populations.^39^ In our implementation of this formula, the average individual living in the most deprived fifth of English small areas was estimated to have 3.8% more need than the average individual living in the least deprived fifth in 2013/2014 (see online supplementary appendix table A2.7). This implied additional needs weight for deprived areas may be an underestimate, for three reasons. First, due to data constraints, we were unable to implement one element of the recommended adjustment: temporary resident status in each age-sex category. Second, the health deprivation domain of the IMD 2010 does not fully capture the burden of multimorbidity, which tends to be greater in deprived populations.^9^ Third, the adjustment is based on workload patterns in the early 2000s. If there were substantial unmet needs for primary care in deprived populations in the early 2000s, the adjustment may underestimate the appropriate level of workload in those populations. This limitation means that we cannot draw firm conclusions about levels of need, and in particular we cannot conclude that socioeconomic inequality in GP supply has now been eliminated. However, we can still conclude that there was a reduction in socioeconomic inequality in GP supply relative to need from 2006/2007 to 2011/2012. To challenge that conclusion, one would have to hypothesise an offsetting increase in relative need for primary care in the most deprived fifth of small areas relative to other areas. This is implausible, for two reasons. First, according to the Carr-Hill formula, relative need for primary care in the most deprived fifth of small areas actually decreased relative to need in the most affluent fifth over the 10-year period of the study, due to gradual changes in age-sex composition between deprivation groups (see online supplementary appendix figure 17.1). Furthermore, it is not plausible that there was a sudden and substantial increase in relative needs in the most deprived fifth of areas between 2006/2007 and 2011/2012 relative to the second most deprived fifth of areas. A second limitation is that the official statistics on GP supply do not include data on the supply of locums.^40^ ^41^ However, growth in the use of GP locums in areas struggling to recruit is unlikely to explain our findings since historically recruitment appears to be more difficult in deprived areas.^42^ ^43^

### Comparison with previous studies

Two previous studies have examined changing patterns of inequality in GP supply relative to need in England using national data. Gravelle and Sutton^22^ examined overall inequality in GP supply between Family Practitioner Committee areas from 1974 to 1990 and between Family Health Service Authority areas from 1990 to 1995. They found substantial and persistent overall inequality, with strong within-area correlation between 1975 and 1995—most of the administrative areas that were ‘underdoctored’ in 1974 were still ‘underdoctored’ in 1995. Goddard *et al* extended this time series by adding the years 1996 to 2006, during which period PCT areas were introduced.^23^ They found that overall variation between administrative areas increased between 1995 and 2006. Both studies concluded that NHS policy had little impact on overall inequality in GP supply, though the second concluded that the abolition of entry controls on ‘overdoctored’ administrative areas in 2002 may have increased overall inequality. Our finding of a reduction in GP supply inequality associated with NHS policy in the late 2000s may seem surprising in the light of these previous findings that inequality in GP supply has not changed much since the 1970s. However, these previous studies are not directly comparable to ours since they examined overall inequality in GP supply between large administrative areas, rather than socioeconomic inequality between small areas. Furthermore, they examined earlier time periods subject to different policy initiatives. For example, the deprivation-weighted capitation payment system introduced in 1990 resulted in complex marginal incentive structures that may have merely shifted GPs from one deprived area to another.^22^ By contrast, the EAPMC programme was specifically targeted at opening new GP practices in deprived areas, involved substantial financial expenditure, and was implemented at a time of vigorous centralised NHS target setting and performance monitoring. Viewed in that light, it is less surprising that this programme succeeded in helping to increase GP supply in deprived areas. Equally, it is perhaps not surprising that socioeconomic inequality started to rise again after the programme was wound down in 2011/2012, as money ran out and practices started to close.

### Meaning of the study: possible explanations and implications for clinicians and policymakers

The reduction in socioeconomic inequality in GP supply was associated with national policy to recruit more GPs in deprived areas of England, as announced in the 2006 White Paper and followed by the EAPMC programme from 2008 to 2011. GP supply relative to need increased from 2006/2007 to 2011/2012 in the group of 38 PCTs that received funding from the EAPMC programme, especially in the most deprived fifth of small areas within those PCTs, while decreasing in other PCTs. The increase in GP supply in deprived small areas appears primarily to have been driven by the opening of new practices, rather than recruitment into existing practices. While inequality has increased again since the end of the EAPMC funding it has not yet reached the levels observed in the early 2000s. However, the ongoing NHS funding squeeze and difficulties in GP recruitment and retention particularly in deprived areas suggest that there is a risk of inequality in GP supply continuing to rise in future years. For example, vacancies in GP training posts are especially high in the North of England, where 29% of training posts were unfilled in August 2014.^44^ Retention of GPs is also a significant problem, with one study suggesting that nearly a third of GPs intend to leave direct patient care within 5 years.^31^

### Unanswered questions and future research

It is not known how much more need for primary care there is in deprived areas relative to affluent areas. Our estimates of this are based on the best available measure of need for primary care: the workload adjustment from the 2007 revision of the Carr-Hill formula for allocating primary care resources. Our figures show that in 2013/2014, the most recent year available, the most deprived fifth of areas received slightly more GP supply relative to need than other areas. However, we cannot conclude from this that ‘prorich’ inequality in GP supply has disappeared since, as explained above, there are good reasons for thinking that the Carr-Hill formula may underestimate need in deprived areas.^39^
